# The impact of teacher socio-emotional competence on student engagement: a meta-analysis

**DOI:** 10.3389/fpsyg.2025.1526371

**Published:** 2025-03-07

**Authors:** Zerihun Ayalew Gebre, Mesfin Molla Demissie, Berhanu Mekonnen Yimer

**Affiliations:** ^1^Institute of Education and Behavioral Sciences, Dilla University, Dilla, Ethiopia; ^2^Department of Policy and Educational Leadership, Institute of Education and Behavioral Sciences, Dilla, Ethiopia; ^3^Department of Curriculum and Instructional Supervision, Institute of Education and Behavioral Sciences, Dilla, Ethiopia

**Keywords:** socio-emotional competence, student engagement, teachers' socio-emotional competence, meta-analysis, student

## Abstract

Teaching requires significant emotional investment to foster students' engagement. However, studies of teachers' socio-emotional competence (SEC) and student engagement are limited. This research compiles available evidence to clarify the overall effect of teachers' SEC on student engagement. By conducting a comparative search across databases, such as PubMed, Scopus, Web of Science, Google Scholar, and Science Direct, the researchers found 31 related research articles published from 2018 to 2023. Of these, 21 papers satisfied the specified inclusion or exclusion criteria and were quantitative. Data analysis was conducted using JASP software. The study found a weak positive correlation between teachers' socio-emotional competence (SEC) and student engagement without publication bias, indicating a significant impact on professional development and overall student engagement. Moreover, the forest plot indicates the statistical significance of teachers' socio-emotional competence in student engagement, based on various studies in the current literature. The study highlights the crucial role of teachers' socio emotional competence in fostering student engagement, thereby promoting healthy and resilient development. To improve teacher socio-emotional competence and student engagement, nested mixed-method designs and integration of training into professional development programs are recommended. Further research should incorporate mediation or moderation into teacher engagement to strengthen this relationship.

## Introduction

Socio-emotional competence (SEC) is vital for teachers' support in increasing student engagement. These competencies pertain to the control that teachers exert on the fundamental socio-emotional processes within the learning environment, the student-teacher relationship, and the progression of learner development (Aldrup et al., 2020; Ozerova et al., 2023). Nonetheless, few studies have examined the relationship between teachers' social-emotional competence and student engagement (Zhang et al., 2022). The study indicates that student engagement exerts a greater positive impact on academic achievement than emotional intelligence (Marta and Ruhendi, 2022). Furthermore, fostering constructive interactions with students, cultivating curiosity, and developing behavior alter student conduct (Alzahrani et al., 2019; Martinsone et al., 2022; Oliveira et al., 2021).

Recent research indicates a significant relationship between teachers' socio-emotional competence and students' academic emotions (Gimbert et al., 2021). Arenas et al. (2023) revealed that teachers demonstrate strong socio-emotional competence, and students' exhibit higher levels of engagement. This emphasizes the importance of socio-emotional competence as an aspect necessary for students' personal and psychological development. Khan et al. (2023) state that teachers play a crucial role in fostering these competencies and providing students with support for engagement. By developing their socio-emotional competence, teachers create an interactive learning atmosphere that further enhances student engagement. Teachers can establish interactive learning environments to support students' engagement. Students' learning approaches, effects, and achievements are linked (Innocent and Opiyo, 2021; Hermana et al., 2021). This confirms that the educational environment supports teachers' socio-emotional competence, which is helpful for professional progress (Wu et al., 2023). On the other hand, the study revealed that indicated a significant relationship between teacher support and student engagement among high school students (Qudsyi et al., 2020). In this context, teachers are crucial in ensuring that students are peaceful, safe, and productive in class and beyond class activities. Teacher and student socio-emotional competence is vital to healthy teacher-student relationships, classroom behavioral regulation, and the ability to cater to students' social and emotional needs for enhancement (Hikmawati et al., 2021). On the other hand, students who can regulate their emotions are more likely to be engaged in their studies, which in turn leads to better achievement (Kwon et al., 2018).

The present study examines teachers' socio-emotional competence and its impact on student engagement; the existing literature has identified numerous shortcomings and disagreements regarding the significance of teachers' socio emotional competence to support students' learning and growth in their conduct, academic achievement, as well as social skills (Dirani et al., 2021). The study found that positive teacher-student interactions had a favorable association with greater school success, and teachers who build strong relationships with students tend to have behavioral competencies. Additionally, culturally relevant practice shows positive relationships with behavioral improvements and enhanced academic participation (Fallon et al., 2022). Furthermore, the study findings revealed a positive and significant relationship between the socio-emotional classroom management of teachers and student engagement (Palarisan and Domag, 2023).

Despite these findings, there is a discrepancy between teachers' socio emotional competence and students' academic interests and performance, which leaves room for additional research (Khan et al., 2023). One of the studies revealed that students were moderately engaged in learning (Yu et al., 2019). Not much is known regarding teachers' socio-emotional relationships with students' academic interests and performance (Zhang et al., 2022). There is limited knowledge of teachers' socio-emotional competencies and student engagement. Additionally, the study indicated that sensed support from teachers positively predicted cognitive learning techniques (Martínez et al., 2023).

The current literature also demonstrates an absence of activity in related contexts and practices that effectively enhance student engagement in educational settings. Increased student participation can be achieved by altering learning environments, enhancing relevance, and building positive interactions (Kassab et al., 2022). Meta-analyses are required to improve understanding of the impact of teachers' socio-emotional competence on student engagement. By determining effect sizes and addressing the heterogeneity shown in earlier research, these analyses offer a more thorough understanding of the relationship between teachers' SEC and student engagement.

## Objectives of the study

Provide insights into the influence of teachers' socio-emotional competence on student engagement in several studies.

Determine the effect sizes of the relationship between teachers' socio-emotional competence and student engagement.

## Research questions

What is the overall effect size of teachers' socio-emotional competence on student engagement?

How do different dimensions of teachers' socio-emotional competence relate to student engagement?

## Methodology

A literature search was conducted to investigate the impact of teachers' socio-emotional competence on student engagement. To identify additional studies, meta-analyses of several literature sources were conducted. The primary purpose was to influence teachers' socio emotional competence and student engagement. The researchers collected and analyzed primary data from multiple sources. A comprehensive literature search covering the 2018–2023 years of the study produced 31 papers on 21 studies that prioritized socio emotional competence, quantitative nature, and student engagement. To minimize bias, internationally published articles published in English that were peer-reviewed were included in the study. The findings provide important new information on the status of research on how teachers affect student engagement.

### PICO framework

To address the aforementioned study problems, the following PICO framework was used.

Population: Teachers and students in a learning environment.

Intervention higher socio-emotional competence among teachers.

Comparison: Comparison of teachers with high vs. low levels of socio-emotional competence.

Outcome: Sustained student engagement.

### Design of the study

**Figure 1:**
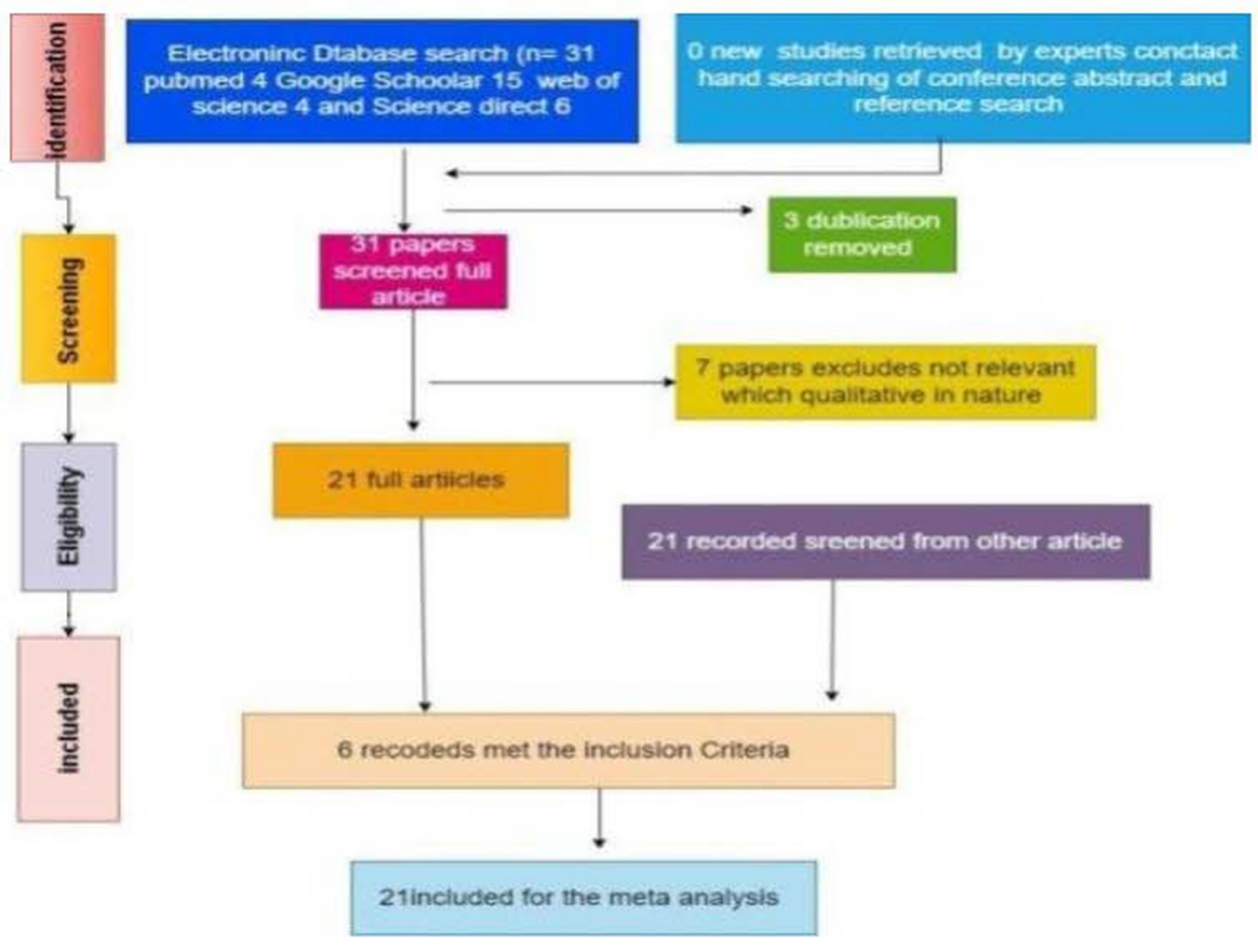
Flowchart of the meta-analysis (PRISMA flow diagram).

This meta-analysis employed the random-effects approach to assess the impact of teachers' socio emotional competence on student engagement. The fixed-effects model failed to accurately represent the true mean of any study because of factors beyond sampling variation. The fixed-effects model gives equal importance to within-study and between-study variations; the random-effects model considers both. A random-effects model captured the true impact of teachers' socio-emotional competence, and the impact of that variable on student engagement may differ across studies because subjects, procedures, or contexts are different. Consequently, the actual impact of teachers' socio emotional competence on student engagement varies significantly across different settings. Using random effects makes the model more complex and minimizes bias by accounting for variations in studies; hence, it diminishes the total effect estimation. Most of the articles related to quantitative research focused on the components of inclusion criteria, specifically teachers' socio emotional competence and student engagement, with attention given to their names and keywords. The research section includes articles published within the 2018–2023 years because of the recent phenomenon of student engagement. The Figure 1 presents the PRISMA diagram for the meta-analysis concerning the impact of teachers socio-emotional competence on student engagement. Table 1 illustrates that all articles used for the metadata representation concerning teachers' socio-emotional competence relate to student engagement. Table 2 indicates the checklist used to conduct the meta-analysis regarding the impact of teachers' socio-emotional competence on student engagement.

**Table 1:** The study includes an article discussing the impact of SEC on student engagement.

**No-**	**Study name**	**Year**	**Variable**	**Mean**	**SD**	**Sample size**	**Standard error**	**Weighted means**	**References**
1	Jiménez-López et al.	2021	Student engagement	2.15	0.73	1,180.00	0.02	2.15	Sureda-García et al., 2021
2	Korbel and Paulus	2018	Autonomy	5.21	68.00	404.00	3.38	0.26	Korbel and Paulus, 2018
3	Rocío Huerta Cuervo et al.	2022	Involvement	4.34	0.62	404.00	0.03	4.34	Huerta Cuervo et al., 2022
4	Novie and Concepcion	2023	Student Engagement	3.98	0.58	377.00	0.03	3.98	
5	Novie and Concepcion	2023	Emotional engagement	4.26	0.17	377.00	0.01	4.26	
6	Baloran et al.	2021	Student engagement	3.98	0.65	529.00	0.03	3.98	Baloran et al., 2021
7	Qiong Liu et al.	2022	Involvement	3.64	0.77	466.00	0.04	3.64	Liu et al., 2022
8	Qian Meng and Qi Zhang	2023	Academic Engagement	3.35	0.91	258.00	0.06	3.35	Meng and Zhang, 2023
9	Pihla Rautanen et al.	2020	Study engagement	4.51	1.41	149.00	0.12	4.51	
10	Pihla Rautanen et al.	2020	School engagement	5.37	1.22	149.00	0.10	5.37	
11	Qin Luo et al.	2023	Learning engagement	4.22	0.92	1,158.00	0.03	4.22	Luo et al., 2023
12	Wang L	2022	Learner engagement	4.86	1.02	365.00	0.05	4.86	Wang, 2022
13	Xuejiao Cheng et al.	2022	Teachers' emotional competence	3.48	0.86	74.00	0.10	3.48	Cheng et al., 2022
14	Qiong Liu et al.	2022	Learning engagement	3.82	0.70	466.00	0.03	3.82	Liu et al., 2022
15	Chernyshenko et al.	2018	Emotional support	4.19	0.78	466.00	0.04	4.19	Chernyshenko et al., 2018
16	Foo & Kutty	2023	Student Engagement	3.77	0.74	351.00	0.04	3.77	
17	Welmilla	2020	Student engagement	3.42	0.62	1,455.00	0.02	3.42	Welmilla, 2020
18	Zhao and Yang	2022	Academic Engagement	76.05	20.03	1,094.00	0.61	76.05	Zhao and Yang, 2022
19	Aldrup et al.	2020	Emotional engagement	24.31	3.17	166.00	0.25	24.31	Aldrup et al., 2020
20	Guo	2021	Learning engagement	0.74	0.65	707.00	0.02	0.74	Guo, 2021
21	Zhiling	2023	Learner engagement	4.05	0.80	1,129.00	0.02	4.05	

**Table 2:** Meta-analysis impact of teachers (SEC) on student academic engagement.

**Questions**	**Assessment**
	**Yes, no, unclear**
Is the review question presented openly and clearly?	Yes, student engagement and teachers' socio-emotional basic competence were associated with the research question.
Were the inclusion criteria appropriate for the review question?	Yes, the teacher variables that are the focus of the inclusion criteria are the direct or indirect effects of teachers' SEC on students' engagement; the variables that are the focus of the inclusion criteria are all the remaining variables related to the teachers' characteristics or to other outcomes that are not the academic achievement of the students.
Was the search strategy appropriate?	Yes, systematic searches of relevant databases and international, national, and regional journals and academic journals, including conference papers and theses, should form part of the search strategy.
Were the resources and sources used to conduct sufficient research?	Yes, considering the limitation of international publication sources and the fact that the sources are published in English, it is necessary to use subject-specific databases and electronic databases (Pubmed, scopus, and web of science).
Were the criteria for appraising studies appropriate?	Yes, study design, methodological issues, sample selection, data analysis or interpretation, and sources of bias should all be key components of the appraisal criteria.
Did two or more reviewers perform the critical evaluation separately?	Yes, to ensure balance and eliminate as much bias as possible, each study should, in an ideal world, be rated by two different scholars.
Are there methods to minimize data extraction errors?	Yes, defined data capture forms and procedures were used in the evaluation to further reduce errors and guarantee accuracy and consistency.
Were the techniques used to appropriately integrate the research?	Yes, the studies and data types included in this meta-analysis were statistically suitable.
Was the likelihood of publication bias assessed?	Yes, the review assessed the extent of publication bias and other statistical approaches, such as Egger's test and funnel plots, to assess the effect.
Did the presented statistics support policy and/or practice recommendations?	Yes, the review used the strength and quality of the evidence to translate the findings into concise, doable recommendations for policy and practice in teacher education and, professional development.

This checklist was based on Aromataris et al. (2015).

### Data analysis

JASP software version 0.18.1.0 was used for the meta-analysis of data format, demonstrating reliable presentation in the data analysis of meta-analysis data type (Azzahrah et al., 2021) and falling within the analytical method in studies in education, making such analysis essential for research. In addition, it is open-access software.

The statistical analysis revealed significant variations in the study results in both the omnibus test of model coefficients and the residual variation test, with a *Q*-score of 5.133 and a *Q*-value of 54,267.161. This indicates substantial outcome variation across studies, validated by two tests showing significant differences. These findings highlight the impact of teachers' socio-emotional competence on student engagement and provide insights into essential factors influencing this relationship. Table 3 depicts the random effect of teachers' socio-emotional competence on student engagement.

**Table 3:** Random effects.

	**Q**	**df**	***p***
Omnibus test of model coefficients	5.133	1	0.023
Residual heterogeneity test	54,267.961	20	<0.001

As shown in Table 4, the predicted intercept coefficient was 8.046 with a standard error of 3.552. The *z*-value corresponding to the coefficient of 2.266 was statistically significant, as indicated by the *p*-value of 0.023. There is a 95% confidence that the actual impact of teachers' socio-emotional competence on student engagement is between 1.085 and 15.07. The confidence interval points to a range in which the participants are 95% sure that the value is real. In this meta-analysis, the values ranged from 1.0. Therefore, teachers' SEC determines student engagement. In addition, the reduction in the prediction coefficient is expected to be high.

**Table 4:** Coefficients.

	**Estimate**	**Standard error**	***z***	***p***	**95% confidence interval**	
					**Lower**	**Upper**
Intercept	8.046	3.552	2.266	0.023	1.085	15.007

Wald test.

The table of statistical analysis shows that there is a substantial degree of fluctuation, and the confidence intervals range from 154.485 to 551.543. Broader inversion intervals are also more variable. Further data gathering, including additional analysis, yielded results similar to those of the 100% forest plot data. The unpredictability of the data, as evidenced by the mean value for *H*^2^, implies variation in the research impact. An *I*^2^ score of 100.000 per cent signifies substantial review heterogeneity, whereas the *H*^2^ estimate represses the total review variation. Almost all studies selected for this meta-analysis have different study designs: cross-sectional, small sample size, and descriptive survey; teachers' SEC; and student engagement with the same concept of a self-report questionnaire. Therefore, this may influence the validity of the conclusion; additional meta-analyses must incorporate longitudinal studies and experimental measures to capture the actual truth of the experience. Table 5 represents the heterogeneity of the estimate concerning teachers' socio-emotional competence and its effect on student engagement.

**Table 5:** Residual heterogeneity estimates.

	**Estimate**	**95% confidence interval**	
		**Lower**	**Upper**
τ^2^	264.349	154.485	551.543
T	16.259	12.429	23.485
*I*^2^ (%)	100.000	99.999	100.000
H^2^	313,607.513	183,272.623	654,316.185

The results indicate that teachers with enhanced socio-emotional competence often foster more student engagement. A covariance score of 12.614 indicates a moderate association between these components and engagement. This indicates that teachers' socio-emotional competence correlates with student engagement. Table 6 demonstrates the parameters of covariance associated with teachers' socio-emotional competence and student engagement.

**Table 6:** Parameter covariance.

	**Intercept**
Intercept	12.614

Kendall's τ test indicates a weak relationship between teachers' SEC and student engagement, demonstrating that there is no publication bias. Teachers' SEC has a weak positive rating, indicating a positive correlation with student engagement. Table 7 shows the rank correlation analysis for asymmetry concerning the impact of teachers' socio-emotional competence on student engagement.

**Table 7:** Rank correlation analysis for asymmetry.

	**Kendall's τ**	***p***
Rank test	0.120	0.462

The statistical significance of 0.749 for the funnel plot asymmetry indicates a substantial result, suggesting the absence of publication bias in the study. This implies that the study's conclusions about teachers' SEC and student engagement are less skewed, thereby improving the credibility of the findings. However, other studies should evaluate these results and estimate sources of bias regarding teachers' SEC and student engagement. Table 8 demonstrates funnel plot asymmetry regarding the impact of teachers' socio-emotional competence on student engagement.

**Table 8:** Egger's test for funnel plot asymmetry.

	***Z***	***p***
Sei	0.320	0.749

Bias: intercept from Egger's regression; a *p*-value of < 0.05.

The measured statistically significant value of 0.050, which corresponds to 5% of the conventional level of statistical significance, strengthens the results. This value is < 0.01, indicating that the observed outcome is not random. The significance of studying teachers' socio-emotional competencies as an absolute necessity impacts learners. Table 9 presents a file drawer analysis of the impact of teachers' socio-emotional competence on student engagement.

**Table 9:** File drawer analysis.

	**Fail-safe *N***	**Target significance**	**Observed significance**
Rosenthal	2.152 × 10^+6^	0.050	<0.001

### Forest plot

**Figure 2:**
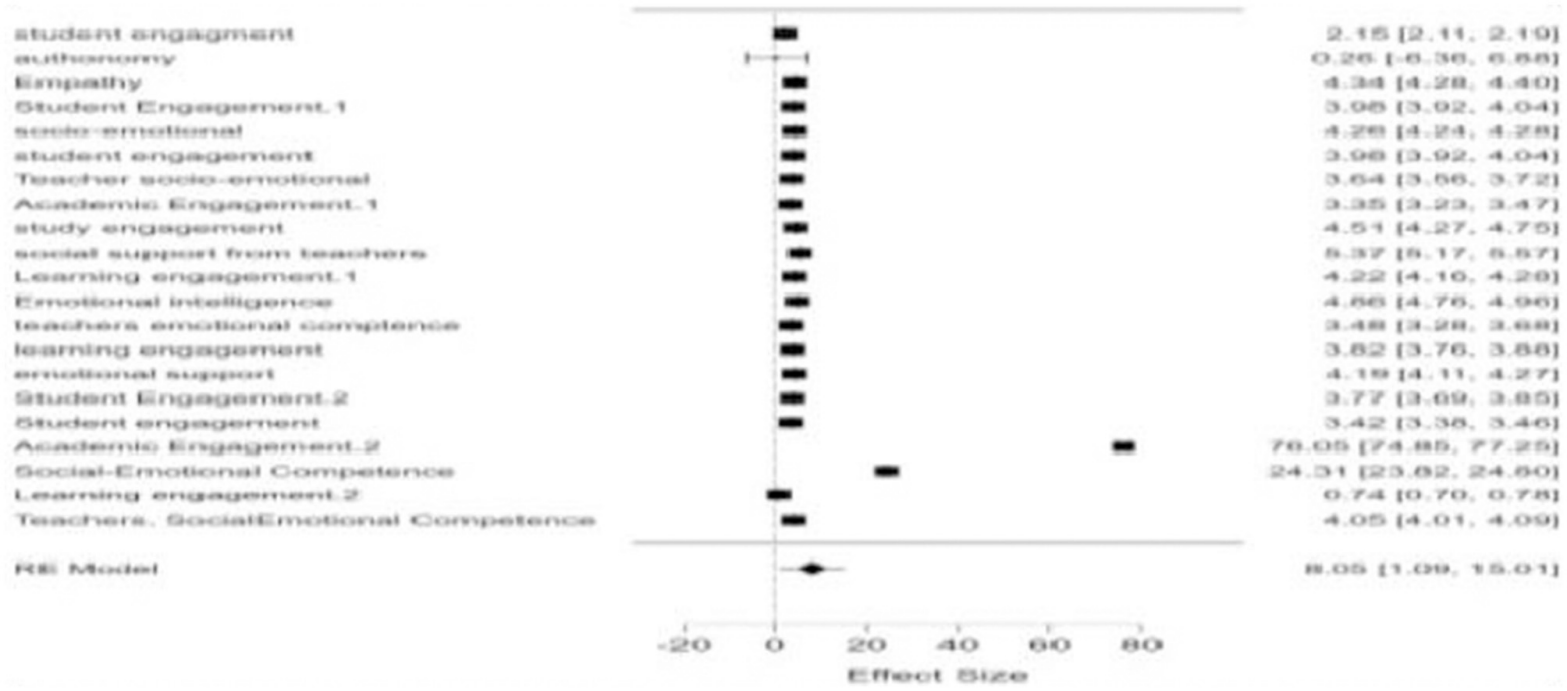
The above plot representation shows the estimated effect of the various investigations incorporated in this meta-analysis. Where the square indicates the effect size, the length of the horizontal line shows the confidence interval for that effect; if the confidence interval crosses the vertical line of no effect (often at 0 for continuous outcomes), it suggests that the study's result is not statistically significant. In general, effect size, represented by a diamond at the bottom of the plot, provides a summary measure of all included studies. If the diamond does not cross the line of no effect, it is then a statistically significant result. Thus, the forest plot revealed significant values indicating that teachers' socio-emotional competence positively influences student engagement across various studies.

The random-effects meta-analysis found significant value in facilitating the influence of teachers' socio-emotional competence on student engagement. The forest plot indicates the effect's level of significance, suggesting that each value in the meta-analysis maintains statistical significance when combined, relating teachers' socio-emotional competence to student engagement from the various studies in the current literature (Figure 2).

### Funnel plot

**Figure 3:**
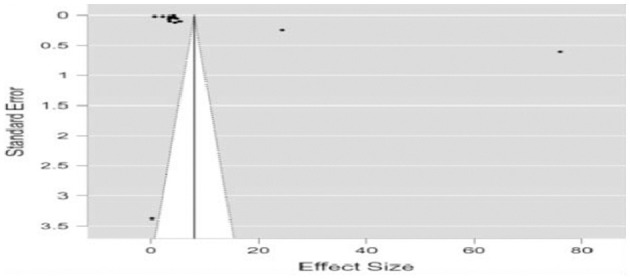
Funnel plot. This funnel plot is used to assess the potential publication biases. The method plots effect sizes from individual studies against a measure of study size or precision (standard error). Smaller studies tend to show more variability; thus, they spread wider apart at the bottom of the funnel. Larger studies are expected to cluster near the average effect size. The asymmetrical funnel plot suggests no significant publication bias. In this analysis, the funnel plot was found to be symmetrical, suggesting no publication bias regarding teachers' socio-emotional competence and its impact on student engagement. This symmetry enhances the credibility of the findings by indicating that the results are not skewed by selective reporting.

Examining the funnel plot, the researcher found that it is symmetrical, and the lack of publication bias has shown how teachers' socio-emotional competence influences student engagement. The arrow represents research either existing in abundance (left side) or its absence (right side of the funnel). Because of this selective publishing, we cannot witness papers from small-scale research projects reporting substandard or erroneous data. Furthermore, the funnel shape of the plot indicates that an even smaller number of articles can be reviewed. Figure 3 displays that the asymmetry of the impact of the teachers socio-emotional competence on the students engagement.

## Discussion

This meta-analysis assesses the impact of teachers' socio-emotional competence and student engagement and demonstrates findings regarding the statistical analysis results and implications for educational practice. The Kendall τ rank test revealed a weak relationship between teachers' socio-emotional competence and student engagement. This indicates that the relationship is not strong between teachers' SEC and student engagement. Furthermore, the random error effect indicates that there is no publication bias at a *p*-value of 0.462, which suggests that the results are accurate. One of the justifications for the weak relationship between teacher socio-emotional competence and student engagement is the relationship between the studied phenomena with characteristics like “weak,” “medium,” “visible,” “high,” and “very high,” fixed alpha level (Igushkin et al., 2022).

This meta-analysis investigates the relationship between teachers' socio emotional competence and student engagement, concentrating only on student engagement, without including sub-constructs like cognitive, emotional, behavioral, and agentic engagement. The study approach was cross-sectional and survey-based, focusing on the elements of socio-emotional competence and student engagement, specifically with teacher-student interactions. This match may reduce the association between the two variables. Future research should examine interventions aimed at enhancing teachers' socio emotional competencies and students' engagement levels.

These findings indicate that teachers' socio-emotional competence regarding absence publishing bias reveals no significant biases or selective reporting in the existing literature to support the claim that this result is typical of the population's effect size. This study highlights that teachers' socio-emotional competence, which is a critical factor in fostering student engagement, plays a vital role in developing healthy and resilient students. Effective professional development is closely linked to years of in-service teaching, experience with social and emotional learning programs, and professional training (García and Gutiérrez, 2022). Additionally, the forest plot supports the rejection of the null hypothesis, demonstrating both significance and effect size in the meta-analysis results. A funnel plot analysis further confirmed the absence of publication bias, which has important implications for the current understanding of teachers' socio-emotional competence and its relationship to student engagement.

Moreover, a higher level of positive socio-emotional competence with similar results indicated that secondary school students from different backgrounds demonstrated that socio-emotional competence was positively correlated with students' engagement and negatively correlated with disengagement, which suggests that educational institutions should invest in social and emotional learning programs to enhance student engagement (Santos et al., 2023; Su Yi and Mydin Kutty, 2023). However, there are at least four limitations to the results. First, the study only included participants from educational settings. Second, the study was conducted using open-access journals, which may have affected the generalizability of the findings. Third, most of the article designs were cross-sectional designs, Lastly, the primary focus of the study is on quantitative research articles, whereas the qualitative results highlight the importance of understanding how instructors' socio-emotional competence influences students' engagement.

Teachers' socio-emotional competence framework differs by country due to the educational system, social structure and cultural value that impact educational learning outcomes and socialization practices. For example, American education tends to emphasize promoting individuality and self-confidence and fostering environments in which self-oriented tasks are valued more highly (Miyamoto et al., 2018). In contrast, Brazil, Vietnam, Mexico, and India are implementing the SEC to address educational inequalities and improve student outcomes, with Brazil's National Standards for Curriculum focusing on cognitive aspects (Cunha et al., 2021). Mexico's SEC is being integrated into education reform efforts through the new model for public education (Bonilla, 2020). In East Asia, such as Japan, socio-emotional competence is more related to social responsibility and group cohesion, which reflects a sociocultural focus on the community. This cultural context shapes educational practices that prioritize collective achievements and interpersonal relationships. In East Africa, particularly Ethiopia, cultural diversity influences emotion and social interactions. Thus, socio emotional competence includes skills such as understanding and managing emotions, empathizing with others, and forming healthy relationships.

## Conclusions

The subsequent conclusion was derived from the meta-analysis results. The forest plots, funnel plot analyses, and Kendall's τ coefficient test validate the primary objectives. Teachers' socio emotional competence significantly influences student engagement and improves learning outcomes. It is essential to prioritize the enhancement of teachers' social-emotional competencies and student engagement. Therefore, it would benefit both teachers and students.

Educational leaders need to be integrated teachers' socio emotional competence into the curriculum to improve student engagement and learning outcomes. Additionally, it is important to design particular strategies that promote teachers' socio emotional competence, which increases student engagement and encourages community and parents' involvement in relation to teachers' socio-emotional competence and student engagement.

To achieve sustainable student engagement, teachers' socio-emotional competence training should be integrated into professional development programs. This training should include the following five socio-emotional competencies of teachers: self-awareness, self-management, social awareness and empathy, relationship skills, and responsible, ethical decision-making. This ensures a strong connection between teachers' and students' engagement in the school, which results in teachers managing stress because the teaching profession is a stressful profession in which students have higher academic performance. Employ specific strategies to enhance socio-emotional competence by including skills training and activities that promote social-emotional learning in the academic curriculum.

Additionally, the results enhance student overall development and support teachers' professional development. However, further study is required to examine how teacher engagement might act as a **moderator or mediator** to improve this association and this research using a longitudinal inquiry design. The research revealed that less robust determinants of teachers engagement in teaching (Valenzuela et al., 2019). Teacher engagement is important because it can link teachers' socio-emotional competence with their beneficial influence on classroom activities and student engagement, promoting a safe atmosphere and improving student relationships. A positive emotional atmosphere in the classroom can foster teachers' socio-emotional competence and student engagement. This harmonious classroom environment fosters healthy student-teacher interactions. Cultural differences can influence the correlation between teachers' (Galugu and Samsinar, 2019) socio-emotional competence and student engagement, as differences in values, beliefs, and attitudes affect the interaction; addressing these factors is essential for enhancing the relationship between teachers' socio-emotional competence. Students' engagement. Additionally, the educational environment can foster teachers' socio-emotional competence in sustained student engagement in learning.

Teachers are engaged emotionally, behaviorally, and cognitively, which enhances students' academic experiences. Higher levels of teachers' engagement can lead to more positive interactions between teachers' socio-emotional competence and students' engagement.

Culturally relevant teaching practices may influence teachers' socio-emotional competence and student engagement in different countries. Different education systems in different countries adopted different curricula and pedagogical experiences, which may invite students' critical thinking and creativity, which can influence teachers' socio-emotional competence and students' engagement. Moreover, family involvement and school discipline practice may influence the relationship between teachers' socio-emotional competence and students' engagement. Future studies should investigate culturally effective and adaptable education practices.

Future research could contribute to existing studies by integrating qualitative analysis that evaluates stakeholder engagement and their perceptions and an attitude regarding the correlation between teachers' socio-emotional competence and student engagement, as the current analysis has a limited scope for quantitative research. Nevertheless, the qualitative assessment of teacher socio-emotional competence and student engagement may provide substantial insight into their interrelationship.

To enhance the relationship between teachers' socio-emotional competence and student engagement, teacher educators and researchers should use concurrent nested mixed-method designs. This should combine both qualitative and quantitative methods, including either teachers' or students' qualitative or quantitative data. and integrating with Kahu's model of student engagement as a theoretical framework.

## Data Availability

The original contributions presented in the study are included in the article/Supplementary material, further inquiries can be directed to the corresponding author.
