# Supplementary figures and images for: The impact of teacher socio-emotional competence on student engagement: a meta-analysis
Source: Front Psychol. 2025 Mar 7;16:1526371. doi: 10.3389/fpsyg.2025.1526371 (PMC11926805; doi:10.3389/fpsyg.2025.1526371)

Appendix

**Diagnostic Plots**


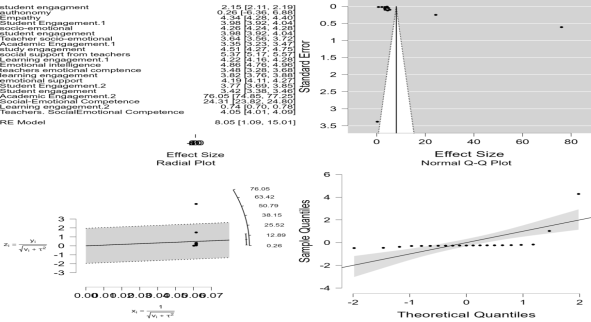


**Log-Likelihood for τ²**


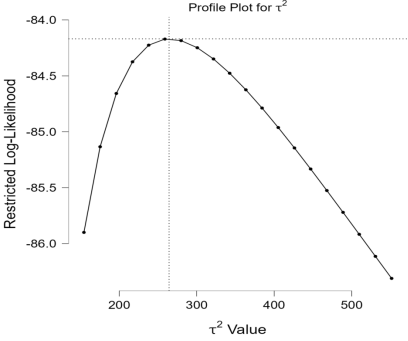

Supplement: Supplementary file 2 [file Table_2.docx]
